# Risk of Multiple Sclerosis in Epstein–Barr Virus Infection

**DOI:** 10.7759/cureus.5699

**Published:** 2019-09-19

**Authors:** Syed Ijlal Ahmed, Kashif Aziz, Amna Gul, Syeda Sana Samar, Syeda Beenish Bareeqa

**Affiliations:** 1 Neurology, Liaquat National Hospital and Medical College, Karachi, PAK; 2 Neurology, Jersey Neurosciences, New Jersey, USA; 3 Internal Medicine, Liaqat National Hospital and Medical College, Karachi, PAK; 4 Internal Medicine, Jinnah Sindh Medical University, Karachi, PAK

**Keywords:** multiple sclerosis, epstein barr virus, infectious mononucleosis, pharyngitis, fever, antibodies, epidemiological studies, virus, ebv, lymphocyte

## Abstract

Multiple sclerosis (MS) is a chronic neuro-inflammatory, immune-mediated disorder of the central nervous system; however, less is known about its cause. It causes neurological disability in young adults, more commonly in women. Several risk factors including environmental, genetics, and infections have been identified, which contribute to the abnormal immune response. Viruses belonging to the Herpes family have been indicated as a potential risk for MS; their biological mechanisms are not known but several possibilities have been discussed. Epstein-Barr virus (EBV) is the leading and most common virus associated with MS. It is a potential oncogenic virus that hosts the B lymphocytes and has been associated with numerous cancers such as Burkitt's lymphoma, Hodgkin's lymphoma, and nasopharyngeal carcinoma. The risk of MS is low in patients who are EBV negative but increases by several folds in individuals who have a history of infectious mononucleosis (IM). Several ecological studies, co-occurring pathologies, and experimental laboratory-based research provide evidence to support the relationship between EBV and MS.

## Introduction and background

In this review, we summarize the evidence associating Epstein-Barr virus (EBV) infection and multiple sclerosis (MS), focusing primarily on the results of epidemiological studies and the presence of antibodies in symptomatic MS patients. EBV is a double-stranded DNA virus that transmits through contaminated saliva and infects the host B-cells. EBV has a diverse clinical presentation; in children, it is often asymptomatic, and in up to 40% adults, it presents as acute infectious mononucleosis (IM) with fever, sore throat, fatigue, and functional impairment, and physical findings include pharyngitis and lymphadenitis [1]. EBV-specific cytotoxic T-lymphocytes and inflammatory cytokines account for the clinical features of IM. Large cross-reactivity of CD 8+ve T-lymphocytes caused by a high viral load or age-related immune response proves the strong correlation of worsening symptoms with age [2]. MS is the most common demyelinating disease and affects 400,000 young adults in America; MS has four disease courses: relapsing-remitting, primary, secondary, and progressive. It presents as a heterogeneous group of symptoms including diplopia, weakness, dyscoordination, sensory loss or distortions, or changes in bowel and bladder function. It is known to be caused by the destruction of the myelin sheath that surrounds the nerve fibers by the innate and adaptive immune system. The innate system plays a role in both the initiation and progression of MS by influencing the effector function of T-cells and B-cells. The presence of EBV in the central nervous system (CNS) and the demonstration of the underlying mechanism associating EBV to the pathogenesis of MS remain to be elucidated [3]. We aimed at understanding the contribution of EBV infection in the pathology of MS.

## Review

Evidence supporting the risk of MS in patients infected with EBV comes from ecological, epidemiological, serological and virological studies. An epidemiolocal study was conducted in England in 2011 to compare the distribution of MS and IM, and a strong correlation was noted in their distribution and the continued existence of a latitude gradient, where MS and IM were noted to be more common in the north than south [4]. Epidemiological reviews in the table below (Table 1) also suggest a strong similarity in the distribution of reported IM and MS [5]. A similar study also describes the latitudinal distribution of MS and infection of EBV throughout the world; its prevalence varies between less than five cases per 100,000 in tropical areas of Asia and greater than 100-200 cases per 100,000 in temperate areas such as Northern Europe, United States, Canada, New Zealand, and Australia. It also describes the influence of several factors on MS such as immigration, Vitamin D, sunlight, and other infectious agents. A study conducted in the UK described the history of patients who acquired oncogenic EBV-induced Hodgkin's lymphoma, who eventually also developed MS [6-8]. These studies also identified multiple risk factors of EBV leading to MS, which includes socioeconomic status, ethnicity, hygiene, and antibodies to EBV nuclear antigen. In western countries, 50% of individuals were found to be infected by EBV during adolescence and young adulthood, increasing the risk by two to three-fold [9].

**Table 1:** Similarities between multiple sclerosis and Infectious mononucleosis epidemiology SES, socioeconomic status; MS, multiple sclerosis; IM, infectious mononucleosis; F, female; M, male; +++, strong evidence; ++, moderate evidence; + weak evidence

Parameter	MS	IM
Age at peak incidence	25-34	15-24
Age at onset	F less than M	F less than M
Extremely rare in tropics	+++	+++
Rare in japan	+++	+++
Rare in Eskimos	++	++
Positive association with SES	+	++
Incidence in blacks	+	+
Incidence in Asians	++	++

The possibilities of an increased risk of MS in developed countries (America and Canada) can be associated with high levels of hygiene for two major reasons. Good hygiene can delay the age of EBV infection (the EBV variant hygiene hypothesis), or the lower load of infections in infancy and childhood can prime the immune system towards a more inflammatory response and increases the risk of autoimmune diseases such as MS [10-11]. Findings from multiple cross-sectional and case-control studies were reviewed; a meta-analysis conducted by Ascherio and Munger described the environmental risk factors attributing to MS. Results showed that the risk of MS in EBV-negative individuals is 15 times lower than that of the EBV positive individuals. More importantly, these results suggest that EBV infection at any age is a major risk factor for MS and cannot be explained by confounding hygiene during childhood [12].

In a large prospective investigation in the American defense forces (the population study), EBV-negative (no detectable anti-EBV antibodies) young adults were directly observed between primary EBV infection and MS occurrence [13]. During the follow-up, seronegative individuals become EBV positive before the onset of MS. The mean interval from when the last seronegative sample was taken to the point of MS symptom occurrence was 5.6 years. Because seronegative participants did not develop MS, the relative risk (RR) of MS following EBV would tend to be infinite. Alternatively, it was also discussed that the pathological process leading to MS starts before the onset of clinical symptoms [14]. The results of this study can therefore explain if pre-clinical MS resulted in increased susceptibility to EBV virus. It was concluded that a positive association existed between EBV and MS and confounding of genetic resistance was ruled out because seronegative patients were eventually infected, thus confirming that EBV is a major risk factor of MS. EBV infection occurred at a rate of about 11% per year, and the incidence of MS increased significantly following EBV infection [13].

Another study discussed the role of human leukocyte antigen (HLA DRB1*1501) haplotype in the development of MS [15]. The authors stated that healthy individuals infected with EBV present with increased risk of MS monotonically by several folds with increasing serum titers of an anti-Epstein-Barr nuclear antigen (EBNA) complex and anti-EBNA-1 antibodies, suggesting possible confounding by genetic factors. Therefore, a case-control study of 148 women was conducted to integrate the relationship between the effects of two different risk factors: EBNA-1 antibody on MS and genetic susceptibility. It was found that both factors independently contributed to increasing MS risk and that the risk among women positive for both factors was nine times higher than that of women with neither. Among healthy women, a correlation between the DR15 allele and anti-EBNA-1 or other antibody titers was not found. As both are independent risk factors, they can be used for screening in first-degree relatives [15]. A meta-analysis of case-control and cohort studies observing the risk of MS following IM conducted by Handel stated the combined RR of MS for a history of IM was 2.17, hence proving a risk of MS following IM [16]. A population-based control study on viral infections, their prevention, and subsequent risk of MS identified the consistent association of MS with IM [9]. Goldacre MJ in a record linkage study noted that previous hospital admissions with IM were associated with a four-fold increase in MS risk compared with a comparison cohort study with a mean interval to MS onset of 14 years. A large cohort study was conducted in Denmark, and IM was diagnosed by the Paul-Bunnell test (titer greater than 1:32), and positive results were associated with increased MS risk (age, sex, and period; standardize incidence ratio = 2.27). In this study, an increased risk of MS appeared 10 years or more after the diagnosis of IM and persisted for more than 30 years [17].

In a prospective nested study of 62439 women, who were followed for years to determine whether elevation in serum antibodies titers to EBV capsid antigen (VCA), nuclear antigen (EBNA, EBNA-1, and EBVA-2), diffuse and restricted early D Antigen (EA-D) and early R Antigen (EA-R) precede the occurrence of MS and its symptoms. 18 cases of MS with blood collected before disease onset, were compared with their matched controls, these women had higher serum geometric mean titers (GMT) of antibodies to EBV but no cytomegalovirus (CMV) (another member of the herpes family). Elevations were significant for antibodies to EBNA-1, EBNA-2, and EA-D. The strongest association was found for antibodies to EBNA-2; a four-fold difference in titers was associated with a relative risk (RR) of MS of 3.9. Significant but generally weaker elevations in anti-EBV antibodies were also found in an analysis of 126 cases of MS with blood collected after disease onset and their matched control [18].

Possible mechanisms of the effect of EBV on MS can be explained by the theory of molecular mimicry between EBV gene products and MS autoantigens [3]. The hypothesis explains that EBV antigen cross-reacts with T-cells that are primed by the exposure, and they cross react with and attack CNS antigens [19]. In support of this, 3% to 4% of EBNA-1 specific CD4 T cells in healthy subjects and MS patients react with peptides derived from myelin proteins, but this does not represent the primary role of EBV in MS pathogenesis. In contradiction to this hypothesis, other infectious agents also have the potential to induce cross-reactivity with CNS antigens (e.g.Gullain Barr virus), but EBV appears to have a unique and persistent role in the development of MS [20,21].

A study by Hassani A was carried out on autopsied brain tissue of patients with clinical symptoms of MS. Polymerase chain reaction (PCR) and EBV-encoded RNA-automated in situ hybridization (EBER-ISH), a very sensitive test, were used for identifying EBV. DNA was extracted from both white matter and meninges to identify the presence of EBV in the brain in most MS cases. This study concluded that EBV is a highly B-cell tropic virus; the findings suggest that in brain tissues of MS cases, cells other than B-cells can also be infected. Almost all of the heavily infected cases in the study showed only limited expression of CD3, CD19, CD20, or CD68. Co-expression of EBER and CD20 in one case with significant inflammatory cellular content suggested that EBV infects CD20-positive B cells. By contrast, cells expressing Glial fibrillary acidic protein (GFAP) and ionized calcium-binding adaptor molecule 1 (Iba1) were more commonly detectable. This study supports a role for EBV infection in MS, as both EBER-ISH and PCR revealed preferential, but scattered and low level of EBV infection in the brain in most MS cases. Thus, without thorough examination, low level of EBV positivity could be easily missed, leading to underestimation of EBV positivity in MS. These data also suggest that EBV may infect more than one cell type in MS, including microglia and astrocytes [22].

## Conclusions

There is strong evidence that EBV and IM precede MS onset. It was proven by multiple studies in various perspectives such as geographical location, viral load, genetic susceptibility, and brain biopsy. The key emerging feature is that EBV affects the humoral and CD8-positive cytotoxic T cells, and dysfunction of these cells in MS is supported by evidence. Epidemiological studies confirm that EBV infection is a strong risk factor for MS development and can be used for screening in high-risk patients. However, the biological mechanisms remain to be well elucidated, although several suggestions have been provided. The epidemiological data also suggest that MS risk could be significantly reduced by preventing EBV infection, which could only be possible with a hypothetical vaccine that develops permanent sterile immunity against EBV. Also, confounding factors such as genetic susceptibility and hygiene were integrated, thereby establishing EBV as an independent risk factor of MS.
